# Dynamic Changes of the Frequency of Classic and Inflammatory Monocytes Subsets and Natural Killer Cells in Chronic Hepatitis C Patients Treated by Direct-Acting Antiviral Agents

**DOI:** 10.1155/2017/3612403

**Published:** 2017-05-08

**Authors:** Gang Ning, Yi-ting Li, You-ming Chen, Ying Zhang, Ying-fu Zeng, Chao-shuang Lin

**Affiliations:** Department of Infectious Diseases, The Third Affiliated Hospital of Sun Yat-Sen University, Guangzhou 510630, China

## Abstract

**Objective:**

Up to now, little was known about the immunological changes of chronic hepatitis C (CHC) patients treated with direct-acting antiviral agents (DAAs); we try to explore the effect of DAAs on the frequency of monocytes, NK cells, and cytokines that promote their activation.

**Methods:**

15 treatment-naive CHC patients and 10 healthy controls were recruited. Patients were examined before DAAs therapy (0 w) and at week 4 (4 w) and week 12 (12 w) of therapy. Percentage of monocytes and NK cells of the peripheral blood was analyzed by flow cytometry. Serum cytokines IL-12, IL-18, CXCL10, CXCL11, sCD14, and sCD163 were measured by enzyme linked immunosorbent assay.

**Results:**

The frequency of CD3^–^CD16^+^CD56^+^ NK cells and classic CD14^++^CD16^−^ monocytes decreased, while CD14^+^CD16^+^ monocytes and cytokines IL-12, IL-18, CXCL10, CXCL11, sCD14, and sCD163 increased at 0 w compared to healthy controls. During DAAs treatment, the decreased NK cells and classic monocytes gradually increased to normal levels; the increased inflammatory monocytes and cytokines IL-12 and CXCL11 decreased to normal levels, but the increased cytokines IL-18, CXCL10, sCD14, and sCD163 still remained at high levels at 12 w though they decreased rapidly from 0 w.

**Conclusion:**

Our results showed that DAAs treatment attenuated the activation of monocytes and NK cells in CHC patients. Trial registration number is NCT03063723.

## 1. Introduction

Treatment of hepatitis C virus (HCV) infection has greatly advanced with the advent of the new direct-acting antivirals (DAAs) in the past 5 years. More than 90% of chronic hepatitis C (CHC) patients could achieve a sustained viral response (SVR) using DAAs after 12 weeks of treatment [1–3]. Among all the DAAs regimens, daclatasvir/sofosbuvir and ledipasvir/sofosbuvir are recommended for all of the genotypes except for patients with genotype 3 infection with cirrhosis by WHO. Now, these two DAAs regimens are included in voluntary licensing agreements signed between the originator companies and generics companies. In fact, daclatasvir/sofosbuvir and ledipasvir/sofosbuvir are already available in generic formulations in some countries. The introduction of generic formulations results in lower prices. It has been report reported that the price for a 12-week regimen of generic sofosbuvir would be less than US$ 500/patient in India, and no doubt, the wide-scale implementation of HCV treatment will be facilitated by this rapid reduction in the price of daclatasvir/sofosbuvir and ledipasvir/sofosbuvir [4].

Previous studies have shown that suppression or eradication of HCV infection has been related to a reduced risk of developing hepatocellular carcinoma (HCC) and improved outcomes in CHC patients [5–7]. In one recent meta-analysis study, the estimated relative risk of HCC development in CHC patients with all stages of fibrosis who achieved SVR by interferon therapy was 0.24, meaning that interferon therapy was able to reduce the risk of HCC occurrence by 76% [8]. Considering high rates of SVR achieved in patients with CHC treated with DAAs, it is reasonable to raise the hope of a drastic decline in HCC occurrence and even a decline in HCC recurrence.

Surprisingly and unexpectedly, increased aggressiveness and high rates of HCC recurrence (28% (16/58) and 29% (17/59), resp.) have been reported in patients who cleared HCV with DAAs after achieving a complete response to resection or local ablation within only 6 months of therapy [9, 10]. The authors hypothesized that the rapid eradication of HCV and control of liver inflammation would impact antitumoral immune control, which in turn might contribute to the neoplastic cells proliferation. Conversely, three independent prospective French cohorts failed to reveal an increased risk of HCC recurrence after DAAs treatment in CHC patients after receiving curative cancer treatments [11]. The conflicting results have raised commentaries and criticism which are a controversial issue with potential clinical implications [12–14].

Although the impact of DAAs treatment on the rate of HCC occurrence or recurrence still remain unclear, it would be more important to pay attention to the immunological changes of CHC patients treated with DAAs. Until now, however, only a few studies were performed to explore the changes of immunological milieu of CHC patients during DAAs treatment [15–17]. Hengst et al. and Carlin et al. explored the effect of DAAs treatment on the inflammatory cytokines and chemokines of CHC patients; they found that DAAs-induced HCV clearance could only partially restore the altered inflammatory mediators [15, 16]. Martin et al. found that DAAs therapy improved the proliferation of HCV-specific T cells, but it still remained unknown to which extent cytokine production of HCV-specific T cells could be recovered [17]. In spite of this, little is known about the influence of DAAs treatment on monocytes.

Here in our study, we aim to explore the effect of antiviral treatment of CHC patients with DAAs on the frequency of monocytes (classic CD14^++^CD16^−^ monocytes and inflammatory CD14^+^CD16^+^ monocytes) [18], NK cells (CD3^−^CD16^+^CD56^+^) [19], and cytokines IL-12, IL-18, CXCL10, and CXCL11, which are necessary to activation of NK cells, and soluble CD14 (sCD14) and soluble CD163 (sCD163), which reflect monocytes activation.

## 2. Material and Methods

### 2.1. Patients and Samples

15 treatment-naive CHC patients (6 males and 9 females) and 10 healthy controls (6 males and 4 females) were recruited at the third affiliated hospital of Sun Yat-Sen university (Guangzhou, China) from January 2016 to November 2016. The mean age of CHC patients was 48.06 ± 3.82 years and the mean age of healthy controls was 27.30 ± 3.40 years. Eight CHC patients were treated with sofosbuvir (400 mg, qd)/ledipasvir (90 mg, qd) for 12 weeks and 7 CHC patients were treated with sofosbuvir (400 mg, qd)/daclatasvir (60 mg, qd) for 12 weeks. Basic characteristics of all the subjects were shown in Table 1. Patients coinfected with HAV, HBV, HDV, HEV, and human immunodeficiency virus were excluded. Besides, pregnant patients or patients with psychiatric disorder were also excluded. This study protocol was approved by the Ethics Review Board of the third affiliated hospital of Sun Yat-Sen university and written informed consent was obtained from the patients before enrollment.

### 2.2. Peripheral Blood Mononuclear Cells (PBMC) Isolation and Storage

All CHC patients were monitored before, during and after DAAs treatment at the out patients clinic of department of infectious diseases. Peripheral blood samples (10 mL) were collected from CHC patients before therapy (0 w), at 4 weeks (4 w), and at the end of treatment (12 w) and from healthy controls with EDTA anticoagulation tubes (Invitrogen, BD). Then PBMC were isolated from peripheral blood samples and cryopreserved at −80°C and 72 hours later were transferred to the liquid nitrogen as previously described [20].

### 2.3. Measurement of Viral Load, ALT, and AST

Serum HCV RNA was quantified by COBAS Taqman assay (Roche Diagnostic, Basel, Switzerland). ALT and AST were measured with Hitachi 7170 automatic biochemistry analyzer in the laboratory center of the third affiliated hospital.

### 2.4. Flow Cytometry

In order to examine the phenotype and frequency of classic CD14^++^CD16^−^ monocytes, nonclassic/intermediate CD14^+^CD16^+^ monocytes and CD3^−^CD16^+^CD56^+^ NK cells, relevant labeled multicolor fluorescence anti-human monoclonal antibodies (mAbs) purchased from eBioscience (San Diego, CA, US) were used for surface staining: anti-CD14-FITC, anti-CD16-PERCP-Cy7, anti-CD3-PERCP, anti-CD56-PERCP-Cy7, and anti-CD16-PE. PBMC was first thawed and then resuspended in flow staining buffer (PBS plus 1% FBS); after being washed twice, PBMC was resuspended again and incubated with the above labeled multicolor fluorescence anti-human monoclonal antibodies. Then the stained PBMC was washed with flow staining buffer and centrifuged. Finally, the stained PBMC were diluted and analyzed on a flow cytometer (BD LSR II) (BD Biosciences). Data was acquired as the fraction of labeled cells within a cell gate set for 20,000 events. The detailed procession was described in our previous study [20].

### 2.5. Enzyme Linked Immunosorbent Assay (ELISA)

Measurement of serum IL-12p70, IL-18, CXCL10, CXCL11, sCD14, and sCD163 were performed with the ProcartaPlex™ Multiplex Immunoassay (eBioscience) according to the manufacturer's protocol using a BD FACSCanto™ II flow cytometer. The Flowlogic™ and Beadlogic™ software (Inivai Technologies, Mentone, Vic., Australia) were used for data analysis.

### 2.6. Statistical Analysis

Normally distributed quantitative data were presented as mean ± standard, while the nonnormally distributed data were expressed as interquartile range. 1-way ANOVA test was used for assessment of the differences among values during the course of treatment, and Mann–Whitney *U* tests or unpaired *t*-test was used for comparison between patients and healthy controls. All data were analyzed by SPSS Statistics 20 and all figures were made by Prizm5.0 statistical analysis software (GraphPad Software). *P* value less than 0.05 was considered to be statistically significant.

## 3. Results

### 3.1. Effect of DAAs Treatment on HCV Viremia and Liver Inflammation

All 15 treatment-naive CHC patients had achieved a rapid virological response (RVR), defined as undetectable HCV RNA <15 U/mL, at the first 4 weeks of sofosbuvir/ledipasvir or sofosbuvir/daclatasvir treatment (Table 1). What is more, none of them experienced virological breakthrough at week 12. Similarly, serum ALT and AST levels had also decreased significantly within the 4 weeks of DAAs treatment and none experienced ALT or AST rebound.

### 3.2. Dynamic Changes of the Frequency of Monocytes Subsets and Natural Killer Cells during DAAs Treatment

**Figure 1:**
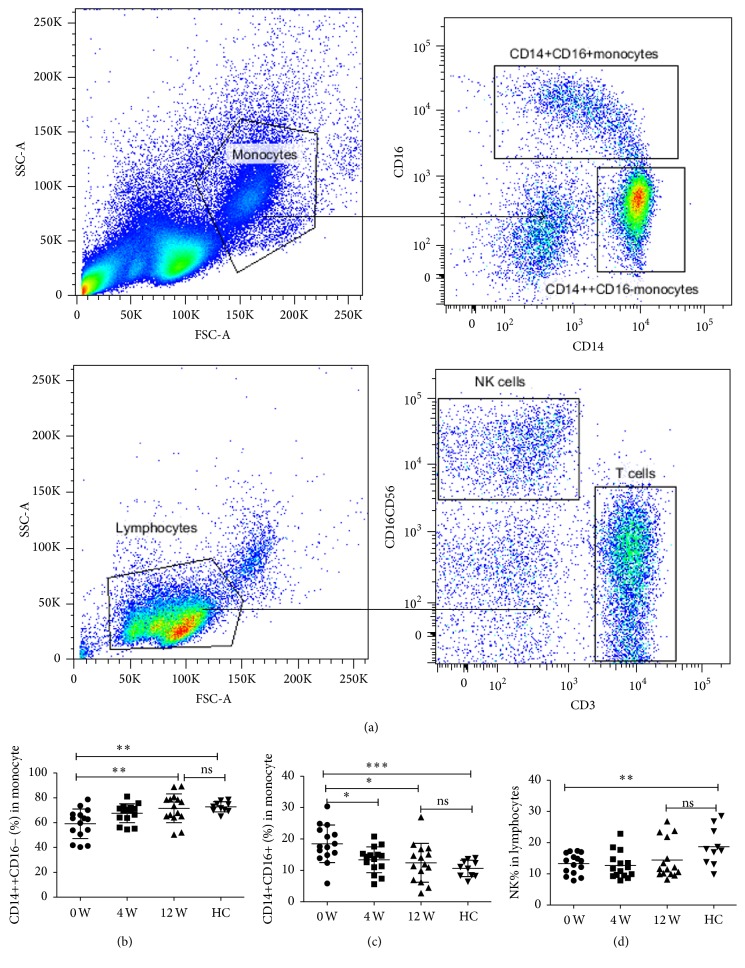
*Dynamic changes of the frequency of monocytes subsets and natural killer cells during DAAs treatment*. (a) Representative flow cytometry plots of CD14^++^CD16^−^ monocytes, CD14^+^CD16^+^ monocytes, and CD3^−^CD16^+^CD56^+^ NK cells; (b) dynamic changes of the frequency of CD14^++^CD16^−^ monocytes; (c) dynamic changes of the frequency of CD14^+^CD16^+^ monocytes; (d) dynamic changes of the frequency of CD3^−^CD16^+^CD56^+^ NK cells. ^*∗*^*p* < 0.05; ^*∗∗*^*p* < 0.01; ^*∗∗∗*^*p* < 0.001.

We used flow cytometry to analyze the dynamic changes of the frequency of monocytes subsets and NK cells in the peripheral blood of CHC patients during DAAs treatment, and representative flow cytometry plots of CD14^++^CD16^−^ monocyte, CD14^+^CD16^+^ monocyte, CD3^−^CD16^+^CD56^+^ NK cells were presented in Figure 1(a). The frequency of classic CD14^++^CD16^−^ monocytes was less than healthy controls at baseline (0 w) (59.14 ± 0.54% versus 72.75 ± 1.31%, *P* < 0.001) and then gradually increased to HC levels (71.54 ± 2.99% versus 72.75 ± 1.31%, *P* > 0.05) during DAAs treatment (12 w). Conversely, the levels of inflammatory CD14^+^CD16^+^ monocyte were higher than HC levels at 0 w (18.49 ± 1.54% versus 10.65 ± 0.83%, *P* < 0.0001) but then rapidly decreased to normal levels of HC (12.42 ± 1.60% versus 10.65 ± 0.83, *P* > 0.05) at 12 w. The changes of the frequency of CD3^−^CD16^+^CD56^+^ NK cells were similar to classic CD14^++^CD16^−^ monocytes. The frequency of CD3^−^CD16^+^CD56^+^ NK cells decreased compared to that of HC level at baseline (13.29 ± 0.85% versus 18.72 ± 1.91%, *P* < 0.001), and after treatment with DAAs, it gradually increased to normal levels of HC at 12 w (14.44 ± 1.60% versus 18.72 ± 1.91, *P* > 0.05) (Figures 1(b)–1(d)). The detailed information about dynamic changes of the frequency of monocytes subsets and natural killer cells was shown in Table 2.

### 3.3. Kinetics of the Levels of Serum sCD14, sCD163, IL-12, IL-18, CXCL10, and CXCL11 during DAAs Treatment

**Figure 2:**
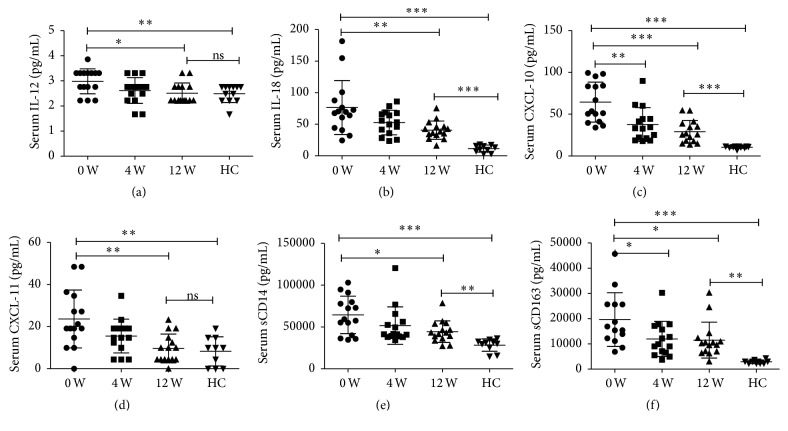
*Kinetics of the levels of serum cytokines during DAAs treatment*. (a) Dynamic changes of the levels of IL-12; (b) dynamic changes of the levels of IL-18; (c) dynamic changes of the levels of CXCL10; (d) dynamic changes of the levels of CXCL11; (e) dynamic changes of the levels of sCD14; (f) dynamic changes of the levels of sCD163. ^*∗*^*p* < 0.05; ^*∗∗*^*p* < 0.01; ^*∗∗∗*^*p* < 0.001.

To further explored the effect of DAAs treatment on the function of monocytes subsets and natural killer cells, we next analyzed the kinetics of the levels of serum sCD14 and sCD163, which reflected monocytes activation and serum IL-12, IL-18, CXCL10, and CXCL11, which promoted NK cells activation during DAAs treatment. Consistent with the above results, all the levels of serum sCD14, sCD163, IL-12, IL-18, CXCL10, and CXCL11 were higher than that of HC at baseline, and during DAAs treatment, sCD14, serum sCD163, and CXCL10 decreased rapidly while IL-12, IL-18, and CXCL11 decreased gradually. However, plasma IL-12 and CXCL11 levels decreased to normal levels of HC, but serum sCD14, sCD163, CXCL10, and IL-18 still remained at high levels (Figures 2(a)–2(f)). The detailed information about dynamic changes of the levels of serum sCD14, sCD163, IL-12, IL-18, CXCL10, and CXCL11 during DAAs treatment was shown in Table 2.

## 4. Discussion

The development of highly effective interferon-free DAAs regiments has revolutionized the treatment of HCV infection and may thus lead to complete eradication of HCV worldwide. However, little was known about the immunological changes of CHC patients during DAAs treatment. So, here in our study, we explored the impact of DAAs treatment on the frequency of monocytes subsets and NK cells.

NK cells are the main innate immune cells of the liver in healthy human and their frequency decreases in the blood but increases in the liver in chronic HCV infection [21]. NK cells from CHC patients are activated by low levels of HCV-induced interferon-*α*, and they expressed increased cytotoxic functions and TNF-related apoptosis-inducing ligand (TRAIL) but decreased antiviral cytokine interferon-*γ* production. Besides, these NK cells also expressed higher levels of activated receptors, such as NKp30, NKp44, NKp46, NKG2C, NKG2D, and CD122, and inhibitory receptor NKG2A than those from healthy controls [22, 23]. Previous studies had explored the effect of interferon therapy on the NK cells. It has been shown that CHC patients with SVR by interferon therapy exhibited greater levels of NK cell degranulation and enhanced NK cytotoxicity and thus, NK cell responses can be used as an indicator of a patient's interferon responsiveness [24, 25]. Recently, Spaan et al. explored the effect of DAAs therapy on NK cells; they found that DAAs therapy increased the percentage of CD3^+^CD56^dim^ NK cells, downregulated surface NKp30, NKp46, and NKG2A expression on NK cells to a phenotype resembling healthy controls, and decreased NK cell-related cytokines (IL-12, IL-18) and TRAIL expression of CHC patients during DAAs treatment [26]. In line with Spaan's study, we also found that NK cells frequency of peripheral blood decreased before DAAs treatment but then gradually increased to normal levels of healthy controls. The changes of serum IL-12, IL-18, CXCL10, and CXCL11 were consistent with the changes of NK cells. They were higher than healthy controls at baseline but then diminished during DAAs treatment. IL-12, IL-18, CXCL10, and CXCL11 are important for NK cells activation. IL-12 and IL-18 could promote interferon-*γ* production of NK cells, and CXCL10 and CXCL11 could activate NK cells to express higher levels of STAT1 and pSTAT1, which are an essential part of signaling downstream of the interferon receptor [27]. Serti et al. demonstrated that DAAs treatment decreased serum levels of CXCL10 and CXCL11, leading to decreased expression of the STAT1 and pSTAT1 of NK cells, and the decreased expression of STAT1 and pSTAT1 was associated with normalization of NK cells phenotype observed in Spaan's study [26, 27]. Furthermore, Serti et al. had also found that DAAs treatment-induced normalization of NK cells phenotype and function may follow a hierarchy. Briefly speaking, significant decrease in HCV titer by DAAs treatment first induced decrease in activation surface HLA-DR expression of NK cells and then reversed the alter cytokine production by NK cells and last normalized the alter cytotoxicity of NK cells.

Similar to NK cells, monocytes are also an important part of the first line of defense against HCV infection. During HCV infection, peripheral blood monocytes are attracted to the liver and differentiate into macrophages and Kupffer cells. Monocytes/macrophages play an important role in initiating the adaptive immune response and influencing the Th1/Th2 polarization by producing excessive inflammatory and immune-modulatory cytokines, such as IL-10 and IL-12. These cytokines may also impair the ability of antigen presenting cells to activate naive T cells and thus help to HCV replication and establish persistent infection [28]. Zheng et al. found that circulating CD14^++^CD16^−^ monocytes decreased while CD14^+^CD16^+^ monocytes increased in CHC patients when compared to HCV spontaneous resolved and healthy controls, and CD14^+^CD16^+^ monocytes were negatively correlated with HCV viremia but PD-L1/CD86 ratio in CD14^+^CD16^+^ monocytes was closely correlated with HCV viremia [29]. Similarly, in our study, we also found that CD14^++^CD16^−^ monocytes decreased but CD14^+^CD16^+^ monocytes increased at baseline in CHC patients. Furthermore, we also found that CD14^++^CD16^−^ monocytes increased to levels of healthy controls while CD14^+^CD16^+^ monocytes decreased to the levels which was similar to healthy controls during DAAs treatment. Up to now, however, little is known about the effect of DAAs treatment on the function of monocytes. In our study, we found that the changes of serum sCD14 and sCD16 which reflected the monocytes activation were similar to the changes of CD14^+^CD16^+^ monocytes, indirectly indicating a decrease in the monocyte activation. Recently, Bility et al. found that HCV-induced M2 macrophages activation was associated with liver fibrosis during HCV infection and supernatant of HCV-infected cells could polarize human monocytes to a M2-like phenotype. What is more, DAAs treatment attenuated M2 macrophages activation and associated liver fibrosis [30]. Interestingly, Gambato et al. found that DAAs treatment did not have obvious effect on the phagocytic and oxidative burst capacity of monocytes in patients with advanced liver fibrosis [31]. Therefore, further studies are needed to explore the impact of DAAs treatment on the changes of the function of monocytes.

There are several limitations in our study. First, the impact of DAAs treatment on the frequency of monocytes and NK cells was only evaluated at the first 12 weeks of treatment and therefore, the long-term effects still remain unknown. However, we would continue the study and explore the long-term effects by DAAs treatment. Second, CHC patients were not treated with the same DAAs regimen and the HCV genotypes were different; this may have an impact on our results. Finally, we did not evaluate the functional status of circulating monocytes and NK cells which could have been associated with the observed decrease in serum sCD14, sCD163, IL-12, IL-18, CXCL10, and CXCL11.

In conclusion, our results show that DAAs treatment attenuated the activation of monocytes and NK cells in CHC patients during DAAs treatment, indicated by decreased levels of sCD14, sCD163, IL-12, IL-18, CXCL10, and CXCL11 and normalization of the frequency of monocytes and NK cells, but the effect of DAAs treatment on their function still needs further research.

## Figures and Tables

**Table 1:** Basic characteristics of subjects.

Index	CHC Patients	Healthy Controls
Number	15	10
Age (y)	48.06 ± 3.82	27.30 ± 3.40
Gender (M/f)	6/9	6/4
ALT (U/L, mean ± SE)	49.29 ± 7.47	NA
AST (U/L, mean ± SE)	52.78 ± 9.39	NA
HCV-RNA (log⁡10 IU/mL)	6.02 ± 0.30	NA
HCV-RNA genotype	1b (42%)/6a (30%)/2a (28%)	NA
Treatment	Sofosbuvir + ledipasvir (8/15), sofosbuvir + daclatasvir (7/15)	NA
RVR/SVR	100%	NA

CHC patients: chronic hepatitis C patients; RVB: ribavirin; RVR: rapid virological response; SVR: sustained virological response.

**Table 2:** The kinetics of the immunological parameters during the treatment of patients with chronic hepatitis C and their comparison with healthy controls.

Parameter	CHC patients			Healthy controls	*P* value				
	0 w	4 w	12 w		0 W–HC	4 W–0 W	12 W–0 W	4 W–12 W	12 W–HC
CD14^++^CD16^−^ monocyte cells (%)	59.14 ± 0.54	67.59 ± 1.98	71.54 ± 2.99	72.75 ± 1.31	*∗∗*	ns	*∗∗*	ns	ns
CD14^+^CD16^+^ monocyte cells (%)	18.49 ± 1.54	13.39 ± 1.07	12.42 ± 1.60		*∗∗∗*	*∗*	*∗*	ns	ns
CD3^−^CD16^+^CD56^+^ NK cells (%)	13.29 ± 0.85	12.68 ± 1.09	14.44 ± 1.60	18.72 ± 1.91	*∗∗*	ns	ns	ns	ns
Serum sCD14 (pg/mL)	64407.38 ± 5778.49	51639.83 ± 5778.49	44390.06 ± 3330.17	28370.76 ± 2357.68	*∗∗∗*	ns	*∗*	ns	*∗∗*
Serum sCD163 (pg/mL)	22853.80 ± 4137.61	11975.35 ± 1795.91	11494.79 ± 1836.97	2934.41 ± 223.31	*∗∗∗*	*∗*	*∗*	ns	*∗∗*
Serum IL-12 (pg/mL)	2.98 ± 0.13	2.61 ± 0.13	2.51 ± 0.10	2.49 ± 0.1	*∗∗*	ns	*∗*	ns	ns
Serum IL-18 (pg/mL)	76.51 ± 11.01	52.73 ± 5.00	40.63 ± 3.72	11.45 ± 1.77	*∗∗∗*	ns	*∗∗*	ns	*∗∗∗*
Serum CXCL-10 (pg/mL)	64.41 ± 6.16	37.64 ± 5.22	29.09 ± 3.49	10.48 ± 0.43	*∗∗∗*	*∗∗*	*∗∗∗*	ns	*∗∗∗*
Serum CXCL-11 (pg/mL)	23.64 ± 3.54	15.53 ± 2.07	9.64 ± 1.77	8.27 ± 2.19	*∗∗*	ns	*∗∗*	ns	ns

CHC patients: chronic hepatitis C patients; ^*∗*^*P* < 0.05; ^*∗∗*^*P* < 0.01; ^*∗∗∗*^*P* < 0.001; ns: no significance.

## Abbreviations

HCV: Hepatitis C virus
DAAs: Direct-acting antivirals
CHC: Chronic hepatitis C
SVR: Sustained viral response
HCC: Hepatocellular carcinoma
RVR: Rapid virological response
TRAIL: TNF-related apoptosis-inducing ligand.
